# Editorial: Multi-Omics Approaches to Study Signaling Pathways

**DOI:** 10.3389/fbioe.2020.00829

**Published:** 2020-09-04

**Authors:** Jyoti Sharma, Lavanya Balakrishnan, Sandeep Kaushik, Manoj Kumar Kashyap

**Affiliations:** ^1^Institute of Bioinformatics, International Technology Park, Bengaluru, India; ^2^Manipal Academy of Higher Education, Manipal, India; ^3^Mazumdar Shaw Center for Translational Research, Bengaluru, India; ^4^3B's Research Group, University of Minho, Guimarães, Portugal; ^5^Amity Medical School, Amity Stem Cell Institute, Amity University Haryana, Gurugram, India

**Keywords:** omics, transcriptomics, proteomics, genomics, microRNA, targeted therapy, signaling

With the advent of omics technologies, tremendous progress has been made in understanding the signaling pathways in normal and disease states across different species. Multi-omics approaches can be categorized into two groups: molecular profiling (MPro) and molecular perturbation (MPer) (Yao et al., 2015). The MPro grouping includes the profiling of genomic, transcriptomic, proteomic, post-translational modifications, and interactome; and MPer includes genetic and functional perturbations.

Omics approaches such as genomics, transcriptomics, miRNAomics, proteomics, and metabolomics have changed the landscape of different diseases including stroke, diabetes, and cancer. Genomic approaches such as genome wide association studies have led to the identification of 30 loci, which were used in swaying body mass index and the risk of obesity (McCarthy, 2010). At mRNA levels, transcriptomic profiling using cDNA microarrays has helped not only in detecting the downregulation of significant tumor suppressors in breast cancer metastasis (Zheng et al., 2017) but also enabled medical practitioners to discriminate patients with activated B-like diffuse large B-cell lymphoma (DLBCL) from those with germinal center B-like DLBCL (Alizadeh et al., 2000). High-throughput studies focused on microRNAs (miRNAs or miRs) in early stage breast cancer have led to the identification of unique predictive miR signatures specific to ER, PR, and HER2 status (Lowery et al., 2009). At the protein level, an *in vivo* labeling technique like stable isotope labeling with amino acids in animal cell culture, coupled with a mass-spectrometry based proteomic approach, has allowed for the comparison of different mutations in lung adenocarcinoma cell lines in relation to EGFR signaling (Guha et al., 2008).

For this special issue, we present a collection of 12 articles, which provide a comprehensive overview of the different biological pathways within the MPro and MPer approaches.

WGS approaches have been extensively used to unravel the different types of genomic alterations in cancer and facilitate understanding of the mutational landscape of cancer genomes. Using WGS, Dr. Bandapalli's group identified candidate predisposing genes in families with a reported recurrence of Hodgkin-lymphoma (HL), a lymphoproliferative malignancy of B-cell origin. These variants were prioritized using an in-house pipeline “FCVPPv2.” The authors used this pipeline along with gene/variant panels based on cancer predisposing genes and variants prioritized in the largest familial HL cohort study reported to date, to identify high penetrance germline variants in the HL families. Furthermore, pathway and network analyses of these variants have provided additional molecular cues relevant to the molecular pathogenesis of HL that may aid the development of targeted therapy and the screening of individuals who are at risk of developing HL (Srivastava et al.).

MicroRNAs play a key role(s) in regulating gene expression via either degradation of a transcript or the inhibition of its translation. Dr. Skoblov's group presents initial insights into the complexity of human microRNA-mRNA interactions. They performed a comprehensive computational analysis on HEK293 and Huh7.5 datasets and reported interesting features of human mRNA interactome, along with more than 46,000 experimentally confirmed mRNA-miRNA duplex regions. As part of this study, they also developed a web-based tool, publicly available at http://score.generesearch.ru/services/mirna/ (Plotnikova et al.).

In addition, Dr. Fang's group performed a comprehensive analysis of hsa-let-7i-5p miRNA in normal and pathogenic fibroblasts and studied its role ranging from controlling cellular phenotype to molecular signaling particularly TGF-β signaling (Zhang et al.).

In a transcriptome study, Dr. Evelo's group performed computational analysis on transcriptomic data derived from cattle breeds with different intramuscular fat deposition to identify pathways that define marbling in beef cattle. A total of 17 pathways were significantly dysregulated between well-marbled vs. lean-marbled beef including MAPK and insulin signaling, and immune response associated pathways (Roudbari et al.).

Dr. Guan's group carried out a genome-wide analysis in gastric cancer and identified 548 and 2,399 differentially methylated sites and lncRNAs, respectively. The lncRNAs were able to discriminate between normal vs. cancerous samples of gastric origin (Song et al.).

Dr. Jolly's group carried out a comprehensive and comparative analysis of methods utilizing different transcriptomics signatures to quantify the status of EMT—a cell biological process involved in cancer metastasis and chemoresistance. They showed that these methods exhibited a concordance among themselves in quantifying the extent of EMT in a given sample and that tumor cells can undergo varying degrees of EMT across tumor types. While any of the three methods can capture the generic trend in the EMT status of a given cell (or population), the multinomial logistic regression EMT scoring method has an additional advantage of being able to predict from the transcriptomic signature of a population, whether it is comprised of “pure” single hybrid E/M cells at the single-cell level, or an ensemble of E and M cell subpopulations (Chakraborty et al.).

Another study, by Dr. Imhof's group in the context of hemolytic disorders, described a new ontology and knowledge graph “HemeKG,” which is publicly available at https://github.com/hemekg/hemekg. This resource assembles heme-specific terms to better categorize, organize, and analyze data on the effects of heme on cell biological and biochemical pathways (Humayun et al.).

In a study on the brain disorder Schizophrenia, Dr. Lane's group used an ensemble boosting predictive framework along with random undersampling, to assess the status of schizophrenia in the population of Taiwan by examining the levels of D-amino acid oxidase protein and its interaction partner, the D-amino acid oxidase activator, in the N-methyl-D-Aspartate receptor pathway, as well as by using melatonin levels in the tryptophan catabolic pathway. They also evaluated the performance of the ensemble boosting algorithm and compared it with other widely used machine learning algorithms, including support vector machine, and multi-layer feedforward neural networks. Notably, they showed that it performs better in distinguishing schizophrenia patients from healthy controls (Lin et al.).

Using an integrative approach, Dr. Domingo-Fernandez and his group demonstrated that the choice of pathway database could impact the results of statistical enrichment analysis and predictive modeling. They also developed an integrative pathway resource called “MPath” which showed that using multiple pathway databases or integrated resources could provide more biologically consistent results and improved prediction performances, as opposed to using equivalent pathways from different databases (Mubeen et al.).

Dr. Zayed's group employed an integrative and systematic bioinformatics in treating ovarian cancer, and identified not only the DEGs involved in the cell cycle, but also the hub genes including core genes (*FZD6, FZD8, CDK2*, and *RBBP8*) strongly linked to OC. A large majority of Frizzled receptors including *FZD6* and *FZD8* were involved in the ß-catenin canonical signaling pathway (Udhaya Kumar, Kumar, Siva, Doss, Zayed et al.).

In a study on the autoimmune disease SLE, Dr. Zayed's group used a high-throughput transcriptomics platform to identify dysregulated signaling pathways. They found that four genes including *EGR1, CD38, CAV1*, and *AKT1* were strongly associated with pathways in SLE (Udhaya Kumar, Kumar, Siva, Doss, Younes et al.).

In a study on *M. tuberculosis*, Dr. Gupta's group studied Rv1915/ICL2a protein as there has been difficulty in harvesting the soluble protein. They overcome this by expression of C-terminal truncated Rv1915/ICL2a in the heterologous host *E. coli* BL21 (DE3) (Antil et al.).

This collective effort brings together studies covering models from prokaryotic to eukaryotic organisms using different omics approaches to delineate either signaling or molecules directly or indirectly related to signaling in cancer and/or other diseases and aberrant conditions.

## Author Contributions

JS coordinated the Research Topic. MKK coordinated the editorial. JS, LB, SK, and MKK contributed to the development of the Research Topic, suggested and invited the participants, and helped with the peer review process. All authors have approved the final version of the editorial.

## Conflict of Interest

The authors declare that the research was conducted in the absence of any commercial or financial relationships that could be construed as a potential conflict of interest.

## Abbreviations

DEGs: differentially regulated genes
DLBCL: diffuse large B-cell lymphoma
EMT: epithelial-Mesenchymal transition
FCVPPv2: familial cancer variant prioritization pipeline
GWAS: genomewide association studies
HL: Hodgkin-lymphoma
miRNA or miR: microRNA
MPro: molecular profiling
MPer: molecular perturbation
NMDAR: N-methyl-D-Aspartate Receptor
SLE: systemic lupus erythematosus
WGS: whole genome sequencing.
